# Pathological and virological insights from an outbreak of European brown hare syndrome in the Italian hare (*Lepus corsicanus*)

**DOI:** 10.3389/fmicb.2023.1250787

**Published:** 2023-10-19

**Authors:** Mariagiovanna Domanico, Patrizia Cavadini, Roberto Nardini, Daniele Cecca, Giovanni Mastrandrea, Claudia Eleni, Valentina Galietta, Lorenzo Attili, Antonella Pizzarelli, Roberta Onorati, Cristina Amoruso, Donatella Stilli, Giulia Pacchiarotti, Francesca Merzoni, Andrea Caprioli, Ida Ricci, Antonio Battisti, Antonio Lavazza, Maria Teresa Scicluna

**Affiliations:** ^1^Istituto Zooprofilattico Sperimentale del Lazio e della Toscana “M. Aleandri” (IZSLT), Rome, Italy; ^2^Istituto Zooprofilattico Sperimentale della Lombardia e dell’Emilia Romagna (IZSLER), Brescia, Italy; ^3^Segretariato Generale della Presidenza della Repubblica—Servizio Tenuta di Castelporziano, Rome, Italy

**Keywords:** European brown hare syndrome, EBHSV, *Lepus corsicanus*, hare, outbreak, *Lagovirus*, virus characterization, Italy

## Abstract

European brown hare syndrome (EBHS) is a highly contagious and fatal viral disease, mainly affecting European brown hares (*Lepus europaeus*). The etiological agent, EBHS virus (EBHSV), belongs to the *Lagovirus* genus within the *Caliciviridae* family. The Italian hare (*Lepus corsicanus*) is endemic to Central-Southern Italy and Sicily and is classified as a vulnerable species. *L. corsicanus* is known to be susceptible to EBHS, but virological data available is scarce due to the few cases detected so far. In this study, we describe the occurrence of EBHS in two free-ranging *L. corsicanus,* found dead in a protected area of Central Italy. The two hares were identified as *L. corsicanus* using phenotypic criteria and confirmed through mitochondrial DNA analysis. Distinctive EBHS gross lesions were observed at necropsy and confirmed by subsequent histological examination. EBHSV was detected in the livers of the two animals initially using an antigen detection ELISA, followed by an EBHSV-specific reverse transcription-PCR, thus confirming the viral infection as the probable cause of death. The EBHS viruses detected in the two hares were identical, as based on blast analysis performed for the VP60 sequences and showed 98.86% nucleotide identity and 100% amino acid identity with strain EBHSV/GER-BY/EI97.L03477/2019, isolated in Germany in 2019. Phylogenetic analysis places our virus in group B, which includes strains that emerged after the mid-1980s. This study supports previous reports of EBHS in *L. corsicanus* and further expands the knowledge of the pathological and virological characteristics of the etiological agent. The ability of EBHSV to cause a fatal disease in the Italian hare represents a serious threat to the conservation of this vulnerable species, especially in populations kept in enclosed protected areas.

## 1. Introduction

European brown hare syndrome (EBHS) is a highly contagious and fatal viral disease mainly affecting European brown hares (*Lepus europaeus*) (Frölich and Lavazza, 2007; Capucci et al., 2021; Fitzner et al., 2022). The disease was known to occur in brown hares since the early 1980s and was diagnosed in free-ranging and farmed hare populations across Europe, including Italy, where it is endemic (Lavazza and Vecchi, 1989; Gavier-Widén and Mörner, 1991; Poli et al., 1991; Duff et al., 1994; Scicluna et al., 1994; Chiari et al., 2014). EBHS generally occurs in an acute form with a high mortality rate, between 90% to 100% in adult animals of naïve populations, strongly impacting on the population dynamics (Wirblich et al., 1994). Acutely affected hares often die suddenly of hepatic failure and necrosis without overt clinical signs (Gavier-Widén and Mörner, 1991; Zanni et al., 1993; Lavazza and Neimanis, 2021; World Organisation for Animal Health (WOAH), 2021). Diffuse hemorrhagic lesions and liver degeneration characterize the acute form of the disease. Hares younger than 2–3 months old are considered resistant to EBHS as they do not manifest clinical signs and mortality, but develop antibodies (Zanni et al., 1993; Edwards et al., 2000; Lavazza and Neimanis, 2021). In endemic areas, virus circulation is correlated to hare population density, animal age and season (Scicluna et al., 1994; Paci et al., 2011; Chiari et al., 2014). Where EBHS is endemic, mortality rates are usually lower due to pre-existing population immunity (World Organisation for Animal Health (WOAH), 2021).

EBHS is transmitted by the oral-faecal route through direct or indirect contact with infected sources. Carnivores, insects, birds, and humans can facilitate virus spread, and insects can act as mechanical vectors, but no reservoir hosts other than lagomorphs have been definitively identified. Indirect transmission occurs through contaminated fomites, including equipment, cages, clothes, vehicles, and utensils, likely occur. Infection via contaminated green forage and vegetation is also possible, and lagomorph carcasses are believed to contribute to the environmental persistence of the virus (World Organisation for Animal Health (WOAH), 2021).

The etiological agent, EBHS virus (EBHSV), belongs to the *Lagovirus* genus of the *Caliciviridae* family and has a single-stranded RNA of positive polarity, approximately 7.5 kb in length (Capucci et al., 1991; Poli et al., 1991; Wirblich et al., 1994). The genome contains two open reading frames (ORFs): ORF1 encodes a polyprotein, which is processed by virus-encoded 3C-like protease (3CLpro) for the release of mature non-structural proteins and the capsid protein VP60; ORF2 encodes a minor structural protein (VP10) (Le Gall et al., 1996). Phylogenetically, two lineages are described: group A, which persisted until 1989, when it apparently disappeared; group B, which appeared in the mid-1980s and contains the most recently detected strains (Lopes et al., 2014). Previous studies showed a high genetic homogeneity of EBHSV strains from different European countries despite their geographical occurrence and time of discovery. These strains are phylogenetically closely related (over 97% homology), likely confirming the slow evolutionary dynamics of this lagovirus species (Fitzner et al., 2021).

The Italian hare (*Lepus corsicanus*) is endemic to Central-Southern Italy and Sicily and is classified as Vulnerable according to the International Union for Conservation of Nature in its Red List (Randi and Riga, 2019). Currently, the most conspicuous populations live in protected areas (Buglione et al., 2020). *L. corsicanus* was included among the lagomorph species susceptible to EBHSV (Lavazza and Neimanis, 2021; World Organisation for Animal Health (WOAH), 2022). Sporadic disease cases and serological evidence of specific antibodies were repeatedly reported in free-ranging *L. corsicanus* from Northern Italy (Guberti et al., 2000; Capucci, personal communication). Nevertheless, extensive genetic identification and characterization of the EBHSV strains detected from Italian hares and genetic species identification of the positive hares have yet to be performed. In addition, *L. corsicanus* is also susceptible to RHDV2 (Camarda et al., 2014), a lagovirus which can cause mortality and lesions overlapping those caused by EBHSV, therefore requiring a differential diagnosis.

The present study aims to describe an outbreak of EBHS involving two free-ranging *L. corsicanus* in a protected area of Central Italy and to investigate the viral phylogenetic relationships to reference EBHSV strains identified previously in Italy and Europe.

## 2. Materials and methods

### 2.1. Hare population and location

**Figure 1:**
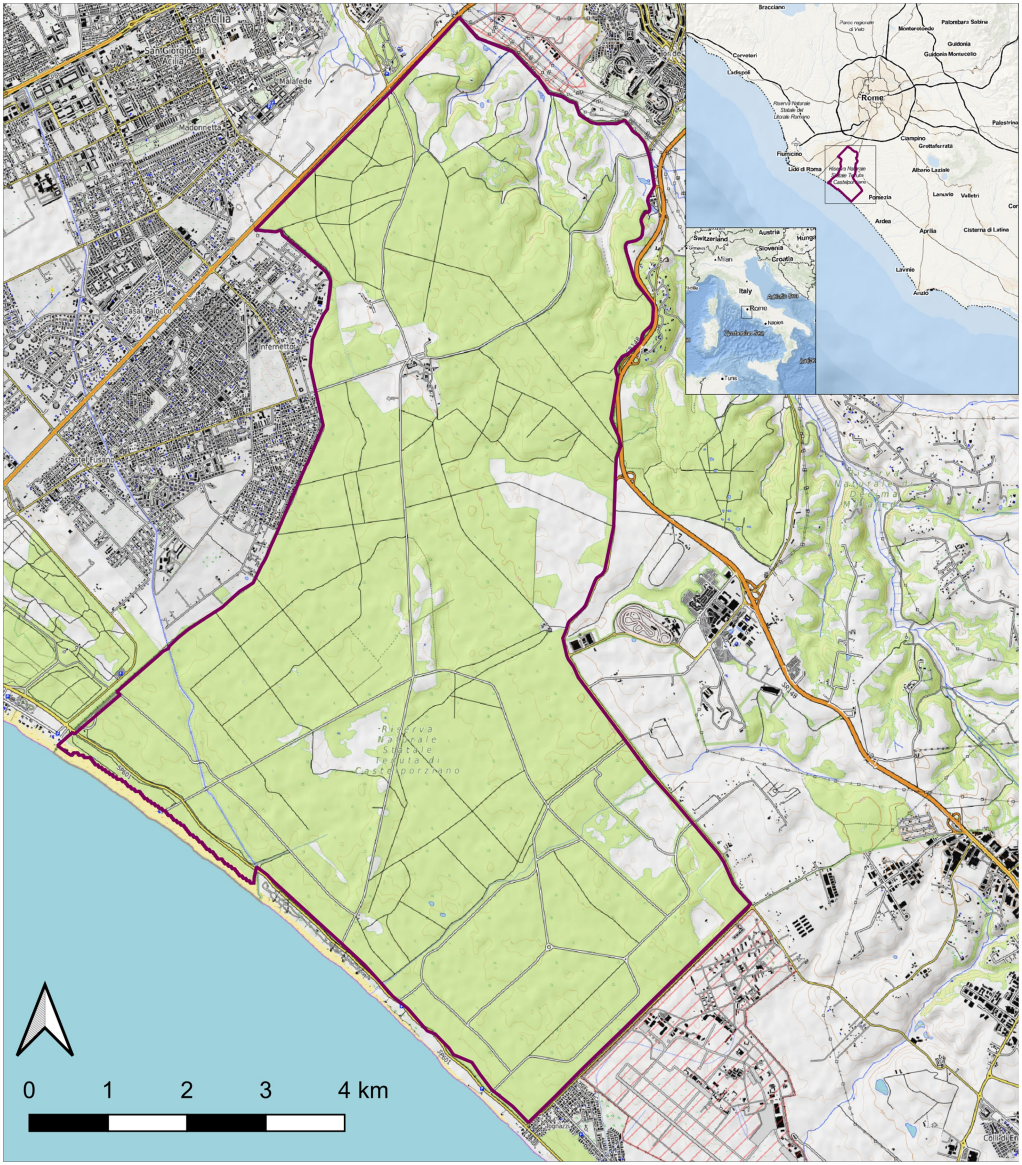
Map of the Castel Porziano Estate, Rome, Italy. At the upper right corner location within Italy and location within the Latium Region are showed. Red lines represent the borders.

In January 2023, two death adult female wild hares (ID: Rm23-1 and Rm23-2), provisionally identified as *L. corsicanus* according to the morphological and phenotypic criteria (Riga et al., 2001; Rugge et al., 2009), were found inside the protected area of Castelporziano Presidential Estate (CPE) (Figure 1). The two dead hares were found 1 week apart in the exact geographical location of the CPE. The CPE is located in Central Italy at around 25 km from the centre of Rome (41°44′037.83″00 N. 12°24′02.20″00 E.) and extends over a surface of 59 km^2^ (5,892 hectares). It englobes most coastal ecosystems typical of the Mediterranean (Latium region, Ecoregion: Mediterranean forests, woodlands, and scrub). Most of the CPE consists of lowland hygrophilous woodlands featuring evergreen and deciduous oak trees and, more specifically, hygrophilous species, especially near the wetlands. Inside the CPE hunting was prohibited since as early as 1977. In 1999, the CPE was assigned the status of Natural State Reserve and subjected to protection measures in line with those protecting natural areas. Regarding hares, the CPE hosts a steady *L. corsicanus* population (latest estimated hare population density of 8.66 hares/km^2^ performed in 2015 based on spotlight counts) with no reported presence of *L. europaeus* (Trocchi and Riga, 2001; Freschi et al., 2022) and apparently no contact with exogenous lagomorph populations. Considering the nature and behaviour of the species involved, although the protected area is fenced, this does not guarantee complete isolation from the brown hare population, which is abundant in the surrounding areas.

Owing to the ongoing research activities, including enhanced passive mortality surveillance of wild animals in the CPE, following the finding of the two hare carcasses, these were submitted to the diagnostic laboratories of the Istituto Zooprofilattico Sperimentale del Lazio e della Toscana “M. Aleandri” (IZSLT) to determine the possible causes of death.

### 2.2. Pathology, sample collection, histopathology, bacteriology and parasitology

A complete necropsy and examination of the two hare carcasses were performed. Samples were taken from several organs, namely liver, lung, trachea, brain, kidney, spleen, heart, uterus, and intestine content; parts were fixed in 10% neutral buffered formalin for histopathological examination, and parts were subjected to standard bacteriological and parasitological analyses. Briefly, for bacteria isolation and identification, tissue/organs were cultured on Columbia agar supplemented with 5% sheep blood (VWR, Belgium) and brain heart infusion broth; following incubation for 24 to 48 h under aerobic and microaerobic (10% CO_2_) conditions at 37°C, growth colonies were subcultured and pure colonies screened by means of standard techniques including colony morphology, Gram staining, catalase test, oxidase test, and biochemically identified at species level with API test kits (bioMérieux, France). Intestine content was screened for the presence of *Salmonella* spp. following international recommendations (World Organisation for Animal Health (WOAH), 2022), as previously described (Alba et al., 2013).

Parasites were screened by macroscopic examination of the intestinal content to detect the presence of proglottids, nematodes and/or fragments of parasites. Samples were subsequently analyzed microscopically by fresh smear and flotation method using saturated NaCl solution (specific gravity 1.2), to evaluate the presence of helminth eggs and/or protozoan (oo) cysts (Riggio et al., 2013).

Livers were also submitted for lagovirus detection by laboratory diagnositic methods, considering the overlapping lesions for RHDV2 and EBHS. Muscle and liver samples were collected to confirm the hare species as *L. corsicanus*, using DNA molecular analyses.

### 2.3. Molecular identification of hares at the species level

Analysis of the mitochondrial control region (CR) was employed to confirm the hare species identification. Total genomic DNA was isolated from tissues (liver, muscle) using QIAamp DNA Mini Kit (Qiagen, Hilden, Germany), following the manufacturer’s instructions. DNA was diluted in 100 μL RNAse-free molecular grade water. A 230 bp fragment in a highly variable region of the mitochondrial CR was amplified through PCR and sequenced (Pierpaoli et al., 1999). Primers were newly designed using the Primer3 web tool^1^ (CR_lepus: F 5′ CATCAGCACCCAAAGCTGAA 3′; R 5′ GCAGGGAATGTGCTATGTCCTA 3′). PCR conditions were the following: an initial activation step at 94°C for 3 min; 38 cycles at 94°C for 30 s, 56°C for 30 s, 72°C for 30 s, followed by a final extension of 5 min at 72°C. Both positive and negative extraction and amplification controls were included. Sequencing was performed using the Applied Biosystems BigDye Terminator Cycle Sequencing Kit (Thermo Fisher Scientific, Waltham, MA, United States), and DNA products were loaded on Applied Biosystems ABI 3130 Genetic Analyzer (Thermo Fisher Scientific, Waltham, MA, United States). Sequences were checked manually and aligned using Geneious Prime 2022^2^ with 15 available online sequences of *L. corsicanus* and *L. europaeus*.

### 2.4. EBHSV detection

Liver homogenates of the two hares were initially analyzed for the presence of lagoviruses, EBHSV and rabbit hemorrhagic disease viruses using a sandwich ELISA followed by a second sandwich ELISA to discriminate between RHDV2 and EBHSV. These methods were set up and validated by the WOAH Reference Laboratory for RHD at the Istituto Zooprofilattico Sperimentale della Lombardia e dell’Emilia Romagna (IZSLER) (Capucci et al., 1991; Le Gall-Reculé et al., 2013; Puggioni et al., 2013; Camarda et al., 2014) and are described in detail in the WOAH Manual of Diagnostic Tests and Vaccines for Terrestrial Animals (World Organisation for Animal Health (WOAH), 2022).

Briefly, the first sandwich ELISA is based on RHDV-EBHSV specific IgG as catcher adsorbed onto the solid phase and a pool of mouse-derived MAbs anti-lagoviruses followed by incubation with rabbit anti-mouse IgG HRP as tracer. The test sample consists of 10% w/v liver extract in phosphate buffer solution, clarified by low-speed centrifugation and tested at two dilutions (1/5 and 1/30) as previously described (Capucci et al., 1991; World Organisation for Animal Health (WOAH), 2022). The second ELISA, the “typing ELISA,” employs a panel of MAbs able to discriminate between RHDV2 or EBHSV (Le Gall-Reculé et al., 2013; Puggioni et al., 2013; World Organisation for Animal Health (WOAH), 2022).

The presence of EBHSV and RHDV2 RNA was also tested by RT-PCR. The total RNA was extracted from the liver using Trizol Reagent (Qiagen, Hilden, Germany), according to the manufacturer’s instructions. Specific RT-PCR for the two viruses (Velarde et al., 2017; World Organisation for Animal Health (WOAH), 2022) were carried out using the Superscript III One-Step RT-PCR kit (Invitrogen, Carlsbad, CA, United States).

### 2.5. EBHSV sequencing and phylogenetic analysis

To determine the VP60 sequences of the field isolates, amplification by reverse transcription (RT)-PCR of four overlapping genome fragments was conducted using primers specific to the EBHSV genome (Table 1). PCR amplicons were visualized in 2% agarose gel, purified (Nucleo Spin PCR and Gel Clean-up, Machery-Nagel, Germany) and directly sequenced in both directions using the ABI Prism BigDye Terminator v3.1 Cycle Sequencing Kit on an ABI 35000XL Genetic Analyzer (Applied Biosystem, Carlsbad, CA, United States). Contigs assembly and genome sequence analysis were performed using Seqman NGen DNASTAR version 11.2.1 (DNASTAR, Madison, WI, United States). For comparative analysis of nucleotide sequences of EBHSV strains, BLASTn software was used (accessed on May 2023). The phylogenetic tree was constructed based on 47 complete VP60 sequences (1731 bp) of lagoviruses by using the maximum likelihood (ML) method and general time reversible model, and the analyses were conducted in MEGA X (Nei and Kumar, 2000; Kumar et al., 2018). Branch support was estimated using 1,000 bootstrap replicates. The phylogenetic relationship among the sequences analyzed was considered reliable when the bootstrap value was higher than 70%.

**Table 1:** Primers used for sequencing the VP60 of EBHSV.

Primers	Sequence(5′-3′)	Position nt^a^	Ta
EBHS-5F	CGACAGGAAGAGGATCGTCT	5,231–5,250	55°C
EBHS-7R	AAACCTGGGGCTGGACCAGC	6,127–6,146	
EBHS-530F	CCTGAAATGTACCACCCAAC	5,793–5,812	52°C
EBHS-911R	CAATGGTGTTGGTTGCACT	6,192–6,210	
EBHS-2F	CTGGAATATGAATGGTGAAACC	6,101–6,122	50°C
EBHS-3R	ATCACCAGTCCTCCGCACCAC	6,666–6,686	
EBHS-1191F	AAGTCGATCTACGGGGTTGCC	6,474–6,494	50°C
EBHS-1786R	GCTCCAGCCAATGTTAGTCCTAGA	7,017–7,040	

a Nucleotide position based on the EBHSV sequence NC_002615.

In addition, to investigate a possible recombination event, the non-structural portion of the EBHSV_Rm23-1 genome was sequenced, and a 5,625 nt fragment was amplified by RT-PCR using HaCV-AF/Rab2 primers (Strive et al., 2009; Cavadini et al., 2021). After purification from agarose gels, the amplicon was subjected to NGS sequencing using the Illumina platform (Illumina, Inc., San Diego, CA, United States) and the full genome was subjected to an RdP4 analysis (Martin et al., 2015).

## 3. Results

### 3.1. Pathology, histopathology, bacteriology and parasitology

**Figure 2:**
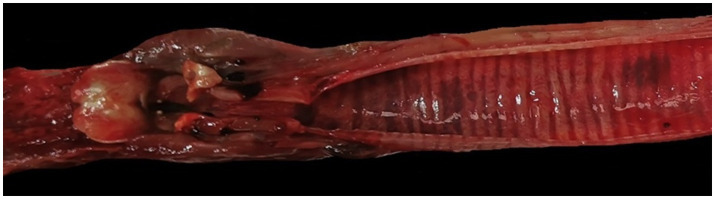
Hare 2. Trachea. Congestion of the tracheal mucosa with multifocal hemorrhages.

**Figure 3:**
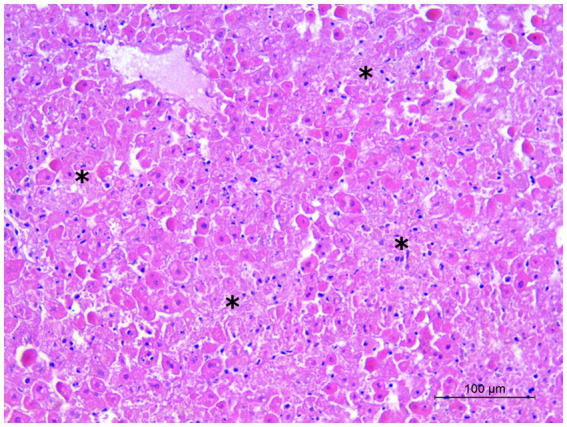
Hare 2. Liver. Multiple foci of hepatocyte necrosis (asterisks). Hematoxylin and eosin stain, 20×.

At necropsy, the hare carcasses presented a good conservation status and body conditions and no apparent external lesions or signs of predation. Macroscopically, the first hare showed blood spilling from the vulva, diffuse congestion and hemorrhages in the tracheal mucosa, mild discolouration and degeneration of hepatic parenchyma, kidney congestion, diffuse congestion, and presence of blood in the left uterine horn. Undigested food was found inside the stomach. The second hare was in lactation and showed blood spilling from the left nostril, tracheal mucosa congestion with multifocal hemorrhages (Figure 2), mild hepatic parenchyma degeneration, splenomegaly, kidneys congestion with cortical petechial hemorrhages and areas of congestion in the left lung. Undigested food was found inside the stomach, showing mild gastric mucosa congestion. Enteric or brain lesions were absent in both animals. The main histopathological finding was liver parenchyma necrosis, associated with periportal hepatitis with lymphoplasmacytic infiltration and rare neutrophils (Figure 3). In addition, kidneys presented diffuse cortical tubulonephrosis, with the formation of multiple small cysts containing amorphous eosinophilic material (a sign of proteinuria), associated with mild membranous glomerulonephritis and vascular congestion. One of the animals (hare ID Rm23-2) also showed areas of vascular congestion in the lung and moderate follicular reactive hyperplastic splenitis, with congested and oedematous red pulp. *Streptococcus gallolyticus* was isolated from the trachea, uterus and intracardiac blood clot and *Acinetobacter lwofii* from the intracardiac blood clot of the first hare. In contrast, for the second hare, all bacteriological cultures were negative. The parasitological examination of the intestine contents performed by flotation detected nematodes and protozoa, namely *Passalurus ambiguus* and *Eimeria* sp. in the first hare, *Trichostrongylus retortaeformis* and *Eimeria* sp. in the second.

### 3.2. EBHSV detection, sequencing and phylogenetic analysis

**Figure 4:**
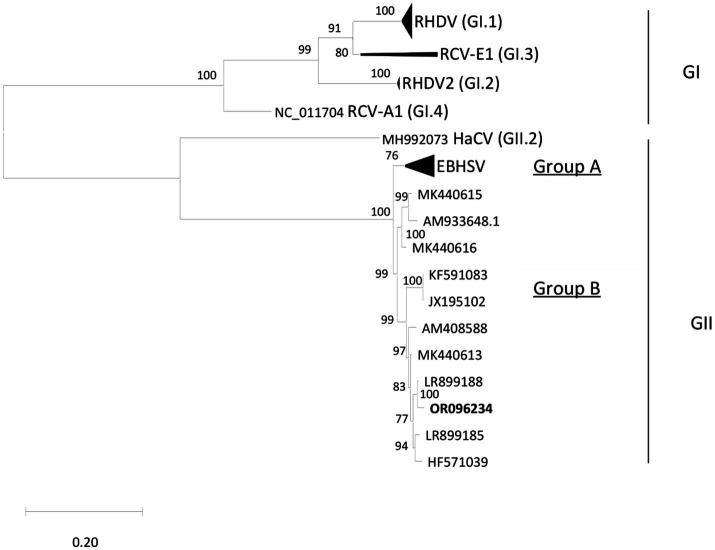
Phylogenetic tree performed with ML-GTR + G + I. The sequence fragments correspond to 47 complete VP60 sequences (1731 bp) of *Lagovirus* retrieved from GenBank. Bootstrap values greater than 70% are shown. Taxa organization of the *Lagovirus* genus (Rabbit *Lagovirus*: GI; Hare *Lagovirus*: GII) is indicated as proposed by Le Pendu et al. (2017).

The livers of both hares tested positive using EBHS sandwich ELISA, and the presence of EBHSV RNA and the absence of RHDV2 were confirmed using the specific RT-PCR amplification. Blast analysis performed on the VP60 (1731 bp) sequences of EBHSV_Rm23-1 and Rm23-2 showed they had a 98.86% nucleotide identity and 100% amino acid identity with the strain EBHSV/GER-BY/EI97.L03477/2019 isolated in Germany in 2019. The phylogenetic analysis conducted on the VP60 sequences showed that the EBHSV_Rm23 strain clustered within the GII.1 genotype, group B, which includes strains that emerged after the mid-1980s (Figure 4). The EBHSV_Rm23-1 genome sequence was deposited in GenBank with accession number OR096234. The RdP4 analysis performed on the full genome sequence of the EBHSV_Rm23-1 revealed no apparent recombination event with other known lagoviruses (data not shown) and showed a 98.56% nucleotide identity with the full genome of EBHSV/GER-BY/EI97.L03477/2019.

### 3.3. Hares genetic identification at the species level

Mitochondrial CR sequences obtained from both hares were identical and clustered within the *L. corsicanus* group. Sequences matched 100% with a haplotype already reported within the *L. corsicanus* species from Central Italy by Pierpaoli et al. (1999) (Genbank Accession number: AF157424).

## 4. Discussion

EBHSV was first detected in European brown hares (*L. europaeus*), and this should be considered the elective host species. However, it is less frequently found also in mountain hares (*L. timidus*) (Lavazza and Vecchi, 1989; Gavier-Widén and Mörner, 1991; Frölich and Lavazza, 2007). The same EBHSV strains can indistinctly infect and cause disease in different hare species, even living in the same areas (Frölich and Lavazza, 2007).

This study describes the molecular characterization of an EBHSV strain detected in two Italian hares (*L. corsicanus*). These results reinforce and complement the already known data regarding the susceptibility of *L. corsicanus* to infection with EBHSV (Guberti et al., 2000) and extend the case history reports of this fatal disease in this species. On the other hand, EBHS was never reported in other European hare species, such as the Iberian hare (*L. granatensis*) and the Broom hare (*L. castroviejoi*), both present mainly in the Iberian Peninsula and in the Cape hare (*L. capensis* var. *mediterraneus*), present in Sardinia (Italy) (Puggioni et al., 2013; Velarde et al., 2017). In addition, field and experimental data demonstrated that the eastern cottontail (*Sylvilagus floridanus*) is susceptible to infection with EBHSV (but not RHDV), occasionally causing EBHS-like disease. So far, infection of the eastern cottontail with EBHSV is considered a spillover event, as this species is deemed a dead-end host, unless new evidence is collected to support an active role in the epidemiology of EBHSV (Lavazza et al., 2015).

In our study, gross pathological and histopathological findings in the two hares investigated were consistent with those described by other authors as typical of lagovirus diseases, mainly characterized by hemorrhages and tracheal mucosa congestion, degeneration of hepatic tissue with necrosis, splenomegaly, lung and kidney congestion (Marcato et al., 1991; Zanni et al., 1993; Gavier-Widén, 1994; Scicluna et al., 1994; Velarde et al., 2017). The good body condition of both animals and the presence of food in the stomach suggest a peracute or acute form of infection. The isolation of bacteria (*Streptococcus gallolyticus* and *Acinetobacter lwofii*) from one of the two hares is probably attributable to secondary infections or post-mortem dissemination, while the detected parasites represent a normal finding in free-living hares. Taken together, acute death, gross and histopathological lesions, positive ELISA and PCR results, in the absence of other evident causes of death, demonstrate that EBHS is the most probable cause of death for both animals. Furthermore, the diagnostic approach used allowed to confirm the presence of a lagovirus in the organs examined, identifying it as EBHSV (EBHSV_Rm23), while ruling out the possibility that it could be RHDV2. This latter virus, which emerged in 2010, is also capable of infecting several other species of hares and other lagomorphs and was repeatedly identified in recent years in Europe, Australia, and the Americas (Puggioni et al., 2013; Camarda et al., 2014; Hall et al., 2017; Velarde et al., 2017; Neimanis et al., 2018; Asin et al., 2021).

The phylogenetic analysis conducted, based on the VP60 sequences, showed that the OR096234 strain belongs to group B, clustering with the GII.1 (EBHSV) genotype, showing 98.86% nucleotide identity and 100% amino acid identity with a strain isolated in Germany in 2019 (Szillat et al., 2020). These findings are consistent with previous VP60 sequence analyses of European EBHSV strains (Lopes et al., 2014; Fitzner et al., 2021), with group B representing the totality of the strains detected recently in Europe, while group A viruses, which encompasses the oldest strains from the Scandinavian Peninsula, disappeared in the late 1980s.When dealing with lagovirus diseases, it is always difficult to trace the origin of the infection, due to the multiplicity of ways, especially indirect, by which the virus is transmitted. Likely, we can rule out that the virus can “persist” in an environment without giving any sign of mortality for years. In fact, to date, the existence of healthy carrier status among lagomorphs or asymptomatic hosts was never demonstrated, as we exclude the cottontail species presence in the CPE, considered as potential spillover species. On the other hand, the virus can also be indirectly transmitted by animated subjects (e.g., predators, both mammals and birds, insects, and humans) and fomites. Considering that in 2023 in Central Italy cases of EBHS were numerous, both in captive and in wild animals, we can consider one of these indirect modes of transmission as the probable source of infection, explaining the epidemiology of this outbreak. Although the entire CPE is fenced, this is unlikely to act as a complete physical separation, considering the epidemiology of lagoviruses and the host species, able to jump over 1.80 meters. It is therefore not possible to prevent contact between the species inside the reserve and those outside, and the possible passage of any EBHSV infection in a context where susceptible species are present. This is precisely the situation that is currently observed in Central-Southern Italy, at least if we refer to the numerous cases recently diagnosed in free-living brown hares (Lavazza, personal communication). It will be interesting to genetically compare our strain with those that have been recently causing cases in Italy, when those sequences will be made available. Our strain is related with a virus lineage consisting of Swedish and French strains discovered from 2008 to 2014, and in particular to newer German strains identified from 2019 to 2020 (Szillat et al., 2020), confirming the successful dispersal and persistence of this lineage throughout Europe (Lopes et al., 2014). High similarities between strains detected in different countries is quite a common evidence for most of the lagoviruses. This is basically due to the low EBHSV genetic variability, and its slow evolutionary dynamics compared with other lagovirus species, such as RHDV (Lopes et al., 2014; Fitzner et al., 2021). Precise definition of the origin of the virus is almost impossible, considering the several ways of transmission described for lagoviruses, however the hypothesis of indirect transmission is the most likely. Data available from some European countries collected during the last 50 years show a drastic decline in the abundance of free-living brown and mountain hares due to environmental and demographic reasons, heavy predation, anthropogenic factors such as poaching, and the occurrence of infectious diseases (Edwards et al., 2000; Smith et al., 2005). In Europe, a constant decline of the *L. europaeus* and *L. timidus* populations was indeed reported due to EBHSV outbreaks (Gavier-Widén and Mörner, 1991; Morisse et al., 1991; Edwards et al., 2000; Fitzner et al., 2021). A threat for hare populations is also represented by RHDV2, which was demonstrated to be able to overcome the interspecies barrier and cause a fatal disease with clinical symptoms similar to EBHS in various hare species, as well as infecting non-lagomorph species (World Organisation for Animal Health (WOAH), 2022). RHDV2 was already reported to infect *L. corsicanus*, although the virulence of RHDV2 is clearly reduced when compared with the far higher mortality observed in *Oryctolagus cuniculus* (Camarda et al., 2014).

*Lepus corsicanus* is classified as vulnerable according to the International Union for Conservation of Nature in its Red List (Randi and Riga, 2019). The distribution of this endemic species was subjected in the last decades to a substantial contraction accompanied by a significant reduction in the population density, mainly due to habitat alterations, low numbers and fragmented populations, and ecological competition with the sympatric European hare (Buglione et al., 2020; Freschi et al., 2022). The occurrence of EBHS in a protected enclosed area, such as the CPE, represents an important threat to the conservation of this vulnerable species that could possibly lead to the point of extinction of the local population. This event would have consequences on the maintenance of the balance and biodiversity in the local wildlife. Although control of the disease in such a situation is difficult, an effort to maintain a disease-free sub-population could be made by isolating some CPE zones or groups of hares combined with the implementation of a vaccination programme, using autovaccines (Drews et al., 2011; World Organisation for Animal Health (WOAH), 2021).

## 5. Conclusion

In this work, we described the anatomo-histopathological lesions and reported the genomic characterization of the EBHSV strain causing an outbreak involving two free-ranging Italian hares (*L. corsicanus*) in a protected area of Central Italy. The identified strain belonged the group B and was phylogenetically related to other EBHSV strains circulating in Europe in *L. europaeus*.

Our findings further confirm that EBHSV can cause fatal disease in the Italian hare, thus representing an important threat to the conservation of this vulnerable species, especially in populations kept in enclosed protected areas.

## Data availability statement

The datasets presented in this study can be found in online repositories. The names of the repository/repositories and accession number(s) can be found at: https://www.ncbi.nlm.nih.gov/genbank/, OR096234.

## Author contributions

AB, AC, CE, MD, MS, PC, and RN: conceptualization. AP, CA, CE, DS, FM, IR, MD, PC, RO, and VG: investigation. DC and GM: resources. MD and PC: data curation. AC, GP, IR, LA, MD, PC, and VG: writing-original draft. AB, AC, AL, CE, MD, MS, PC, and RN: writing-review and editing. MD, PC, RN, DC, GM, CE, VG, LA, AP, RO, CA, DS, GP, FM, AC, IR, AB, AL, and MS: visualization. AB, AL, and MS: supervision. AB, AC, AL, MS, PC, and RN: project administration. AC and DC: funding acquisition. All authors contributed to the article and approved the submitted version.

## Footnotes

^1^ https://primer3.ut.ee/

^2^ https://www.geneious.com

## References

[ref1] Alba P. Terracciano G. Franco A. Lorenzetti S. Cocumelli C. Fichi G.. (2013). The presence of *Brucella ceti* ST26 in a striped dolphin (*Stenella coeruleoalba*) with meningoencephalitis from the Mediterranean Sea. Vet. Microbiol. 164, 158–163. doi: 10.1016/j.vetmic.2013.01.023, PMID: 23419820

[ref2] Asin J. Nyaoke A. C. Moore J. D. Gonzalez-Astudillo V. Clifford D. L. Lantz E. L.. (2021). Outbreak of rabbit hemorrhagic disease virus 2 in the southwestern United States: first detections in southern California. J. Vet. Diagn. Investig. 33, 728–731. doi: 10.1177/10406387211006353, PMID: 33797311 PMC8229834

[ref3] Buglione M. Petrelli S. de Filippo G. Troiano C. Rivieccio E. Notomista T.. (2020). Contribution to the ecology of the Italian hare (*Lepus corsicanus*). Sci. Rep. 10:13071. doi: 10.1038/s41598-020-70013-1, PMID: 32753640 PMC7403147

[ref4] Camarda A. Pugliese N. Cavadini P. Circella E. Capucci L. Caroli A.. (2014). Detection of the new emerging rabbit haemorrhagic disease type 2 virus (RHDV2) in Sicily from rabbit (*Oryctolagus cuniculus*) and Italian hare (*Lepus corsicanus*). Res. Vet. Sci. 97, 642–645. doi: 10.1016/j.rvsc.2014.10.008, PMID: 25458493

[ref5] Capucci L. Cavadini P. Lavazza A. (2021). “Rabbit hemorrhagic disease virus and European brown hare syndrome virus (*Caliciviridae*)” in Encyclopedia of virology. eds. Bamford D. H. Zuckerman M.. 4th ed (Elsevier, Amsterdam, NL: Academic Press), 724–729.

[ref6] Capucci L. Scicluna M. T. Lavazza A. (1991). Diagnosis of viral haemorrhagic disease of rabbits and the European brown hare syndrome. Rev. Sci. Tech. 10, 347–370. doi: 10.20506/rst.10.2.561 1662098

[ref7] Cavadini P. Molinari S. Merzoni F. Vismarra A. Posautz A. Alzaga Gil V.. (2021). Widespread occurrence of the non-pathogenic hare calicivirus (HaCV *Lagovirus* GII.2) in captive-reared and free-living wild hares in Europe. Transbound. Emerg. Dis. 68, 509–518. doi: 10.1111/tbed.13706, PMID: 32603021 PMC8247275

[ref8] Chiari M. Ferrari N. Giardiello D. Avisani D. Zanoni M. Alborali G. L.. (2014). Temporal dynamics of European brown hare syndrome infection in Northern Italian brown hares (*Lepus europaeus*). Eur. J. Wildl. Res. 60, 891–896. doi: 10.1007/s10344-014-0856-6

[ref9] Drews B. Szentiks C. A. Roellig K. Fickel J. Schroeder K. Duff J. P.. (2011). Epidemiology, control and management of an EBHS outbreak in captive hares. Vet. Microbiol. 154, 37–48. doi: 10.1016/j.vetmic.2011.06.021, PMID: 21831541

[ref10] Duff J. P. Chasey D. Munro R. Wooldridge M. (1994). European brown hare syndrome in England. Vet. Rec. 134, 669–673. doi: 10.1136/vr.134.26.669, PMID: 7941275

[ref11] Edwards P. Fletcher M. Berny P. (2000). Review of the factors affecting the decline of the European brown hare, *Lepus europaeus* (Pallas, 1778) and the use of wildlife incident data to evaluate the significance of paraquat. Agric. Ecosyst. Environ. 79, 95–103. doi: 10.1016/S0167-8809(99)00153-X

[ref12] Fitzner A. Kwit E. Niedbalski W. Bigoraj E. Kęsy A. Rzeżutka A. (2021). Phylogenetic analysis of European brown hare syndrome virus strains from Poland (1992–2004). Viruses 13:1999. doi: 10.3390/v13101999, PMID: 34696431 PMC8539919

[ref13] Fitzner A. Niedbalski W. Kęsy A. Rataj B. Flis M. (2022). European brown hare syndrome in Poland: current epidemiological situation. Viruses 14:2423. doi: 10.3390/v14112423, PMID: 36366520 PMC9698305

[ref14] Freschi P. Fascetti S. Riga F. Rizzardini G. Fortebraccio M. Ragni M.. (2022). Diet selection by the Italian hare (*Lepus corsicanus de Winton*, 1898) in two protected coastal areas of Latium. Animals 12:687. doi: 10.3390/ani12060687, PMID: 35327084 PMC8944817

[ref15] Frölich K. Lavazza A. (2007). “European brown hare syndrome” in Lagomorph biology. eds. Alves P. C. Ferrand N. Hacklaender K. (Berlin: Springer), 253–262.

[ref16] Gavier-Widén D. (1994). Morphologic and immunoistochemical characterization of the hepatic lesions associated with European brown hare syndrome. Vet. Pathol. 31, 327–334. doi: 10.1177/030098589403100305 8053127

[ref17] Gavier-Widén D. Mörner T. (1991). Epidemiology and diagnosis of the European brown hare syndrome in Scandinavian countries: a review. Rev. Sci. Tech. 10, 453–458. doi: 10.20506/rst.10.2.555, PMID: 1760585

[ref18] Guberti V. De Marco M. A. Riga F. Cavazza A. Trocchi V. Capucci L.. (2000). Virology and species conservation: the case of EBHSV and the Italian hare. Proceedings of V International Congress of European Society for Veterinary Virology (Brescia, August 27–30, 2000) 198–199. Available at: https://www.researchgate.net/publication/372389197_Virology_and_species_conservation_the_case_of_EBHSV_and_the_Italian_Hare_Lepus_corsicanus_Proceedings_of_the_5_Congress_of_the_European_Society_for_Veterinary_Virology_ESVV_Brescia_27-30_agosto_2000_p

[ref19] Hall R. N. Peacock D. E. Kovaliski J. Mahar J. E. Mourant R. Piper M.. (2017). Detection of RHDV2 in European brown hares (*Lepus europaeus*) in Australia. Vet. Rec. 180:121. doi: 10.1136/vr.104034, PMID: 28154218

[ref20] Kumar S. Stecher G. Li M. Knyaz C. Tamura K. (2018). MEGA X: molecular evolutionary genetics analysis across computing platforms. Mol. Biol. Evol. 35, 1547–1549. doi: 10.1093/molbev/msy096, PMID: 29722887 PMC5967553

[ref21] Lavazza A. Cavadini P. Barbieri I. Tizzani P. Pinheiro A. Abrantes J.. (2015). Field and experimental data indicate that the eastern cottontail (*Sylvilagus floridanus*) is susceptible to infection with European brown hare syndrome (EBHS) virus and not with rabbit haemorrhagic disease (RHD) virus. Vet. Res. 46:13. doi: 10.1186/s13567-015-0149-4, PMID: 25828691 PMC4337088

[ref22] Lavazza A. Neimanis A. (2021). EWDA network for wildlife health surveillance in Europe diagnosis card *Lagovirus* diseases: European brown hare syndrome and rabbit haemorrhagic disease. (EWDA). (Accessed March 21, 2023)

[ref23] Lavazza A. Vecchi G. (1989). Osservazioni su alcuni episodi di mortalità nella lepre: evidenziazione al microscopio elettronico di una particella virale: nota preliminare. Sel. Vet. 30, 461–468.

[ref24] Le Gall G. Huguet S. Vende P. Vautherot J. F. Rasschaert D. (1996). European brown hare syndrome virus: molecular cloning and sequencing of the genome. J. Gen. Virol. 77, 1693–1697. doi: 10.1099/0022-1317-77-8-1693, PMID: 8760416

[ref25] Le Gall-Reculé G. Lavazza A. Marchandeau S. Bertagnoli S. Zwingelstein F. Cavadini P.. (2013). Emergence of a new lagovirus related to rabbit haemorrhagic disease virus. Vet. Res. 44:81. doi: 10.1186/1297-9716-44-81, PMID: 24011218 PMC3848706

[ref26] Le Pendu J. Abrantes J. Bertagnoli S. Guitton J. S. Le Gall-Reculé G. Lopes A. M.. (2017). Proposal for a unified classification system and nomenclature of lagoviruses. J. Gen. Virol. 98, 1658–1666. doi: 10.1099/jgv.0.000840, PMID: 28714849

[ref27] Lopes A. M. Capucci L. Gavier-Widén D. Le Gall-Reculé G. Brocchi E. Barbieri I.. (2014). Molecular evolution and antigenic variation of European brown hare syndrome virus (EBHSV). Virology 468-470, 104–112. doi: 10.1016/j.virol.2014.08.002, PMID: 25155199

[ref28] Marcato P. S. Benazzi C. Vecchi G. Galeotti M. Della Salda L. Sarli G.. (1991). Clinical and pathological features of viral haemorrhagic disease of rabbits and the European brown hare syndrome. Rev. Sci. Tech. 10, 371–392. doi: 10.20506/rst.10.2.560, PMID: 1760582

[ref29] Martin D. P. Murrell B. Golden M. Khoosal A. Muhire B. (2015). RDP4: detection and analysis of recombination patterns in virus genomes. Virus Evol. 1:vev 003. doi: 10.1093/ve/vev003, PMID: 27774277 PMC5014473

[ref30] Morisse J. P. Le Gall G. Boilletot E. (1991). Hepatitis of viral origin in Leporidae: introduction and aetiological hypotheses. Rev. Sci. Tech. 10, 269–310. doi: 10.20506/RST.10.2.549 1760579

[ref31] Nei M. Kumar S. (2000) Molecular evolution and phylogenetics. New York: Oxford University Press.

[ref32] Neimanis A. S. Ahola H. Pettersson U. L. Lopes A. M. Abrantes J. Zohari S.. (2018). Overcoming species barriers: an outbreak of *Lagovirus europaeus* GI.2/RHDV2 in an isolated population of mountain hares (*Lepus timidus*). BMC Vet. Res. 14:367. doi: 10.1186/s12917-018-1694-7, PMID: 30477499 PMC6258167

[ref33] Paci G. Lavazza A. Ferretti M. Bagliacca M. (2011). Relationship between anti-European brown hare syndrome serological titers and brown hare (*Lepus europaeus Pallas*) densities. Int. J. Zool. 2011, 1–5. doi: 10.1155/2011/436193

[ref34] Pierpaoli M. Riga F. Trocchi V. Randi E. (1999). Species distinction and evolutionary relationships of the Italian hare (*Lepus corsicanus*) as described by mitochondrial DNA sequencing. Mol. Ecol. 8, 1805–1817. doi: 10.1046/j.1365-294x.1999.00766.x 10620225

[ref35] Poli A. Nigro M. Gallazzi D. Sironi G. Lavazza A. Gelmetti D. (1991). Acute hepatosis in the European brown hare (*Lepus europaeus*) in Italy. J. Wildl. Dis. 27, 621–629. doi: 10.7589/0090-3558-27.4.621 1661785

[ref36] Puggioni G. Cavadini P. Maestrale C. Scivoli R. Botti G. Ligios C.. (2013). The new French 2010 rabbit hemorrhagic disease virus causes an RHD-like disease in the Sardinian Cape hare (*Lepus capensis mediterraneus*). Vet. Res. 44:96. doi: 10.1186/1297-9716-44-96 24099575 PMC3853023

[ref37] Randi E. Riga F. (2019). *Lepus corsicanus*. The IUCN Red List of Threatened Species 2019: e.T41305A2952954. Available at: 10.2305/IUCN.UK.2019-2.RLTS.T41305A2952954.en. (Accessed March 21, 2023)

[ref38] Riga F. Trocchi V. Randi E. Toso S. (2001). Morphometric differentiation between the Italian hare (*Lepus corsicanus De Winton*, 1898) and the European brown hare (*Lepus europaeus Pallas*, 1778). J. Zool. 253, 241–252. doi: 10.1017/S0952836901000218

[ref39] Riggio F. Mannella R. Ariti G. Perrucci S. (2013). Intestinal and lung parasites in owned dogs and cats from Central Italy. Vet. Parasitol. 193, 78–84. doi: 10.1016/j.vetpar.2012.11.026, PMID: 23265188

[ref40] Rugge C. Mallia E. Perna A. Trocchi V. Freschi P. (2009). First contribute to the characterization of coat in *Lepus corsicanus* and *Lepus europaeus* by colorimetric determinations. Ital. J. Anim. Sci. 8, 802–804. doi: 10.4081/ijas.2009.s2.802

[ref41] Scicluna M. T. Lavazza A. Capucci L. (1994). European brown hare syndrome in northern Italy: results of a virological and serological survey. Rev. Sci. Tech. 13, 893–904. doi: 10.20506/rst.13.3.801, PMID: 7949361

[ref42] Smith R. K. Jennings N. V. Harris S. (2005). A quantitative analysis of the abundance and demography of European hares *Lepus europaeus* in relation to habitat type, intensity of agriculture and climate. Mammal Rev. 35, 1–24. doi: 10.1111/j.1365-2907.2005.00057.x

[ref43] Strive T. Wright J. D. Robinson A. J. (2009). Identification and partial characterisation of a new *Lagovirus* in Australian wild rabbits. Virology 384, 97–105. doi: 10.1016/j.virol.2008.11.004, PMID: 19049842

[ref44] Szillat K. P. Höper D. Beer M. König P. (2020). Full-genome sequencing of German rabbit haemorrhagic disease virus uncovers recombination between RHDV (GI.2) and EBHSV (GII.1). Virus Evol. 6:veaa 080. doi: 10.1093/ve/veaa080, PMID: 33324492 PMC7724246

[ref45] Trocchi V. Riga F. (2001). “Piano d’azione nazionale per la Lepre italica (*Lepus corsicanus*)” in Quaderni di Conservazione della Natura. eds. Trocchi V. Riga F. (Roma: Min. Ambiente-Ist. Naz. Fauna Selvatica), 1–108.

[ref46] Velarde R. Cavadini P. Neimanis A. Cabezón O. Chiari M. Gaffuri A.. (2017). Spillover events of infection of brown hares (*Lepus europaeus*) with rabbit haemorrhagic disease type 2 virus (RHDV2) caused sporadic cases of an European brown hare syndrome-like disease in Italy and Spain. Transbound. Emerg. Dis. 64, 1750–1761. doi: 10.1111/tbed.12562, PMID: 27615998 PMC5697611

[ref47] Wirblich C. Meyers G. Ohlinger V. F. Capucci L. Eskens U. Haas B.. (1994). European brown hare syndrome virus: relationship to rabbit hemorrhagic disease virus and other caliciviruses. J. Virol. 68, 5164–5173. doi: 10.1128/JVI.68.8.5164-5173.1994, PMID: 7518531 PMC236460

[ref48] World Organisation for Animal Health (WOAH). (2021). European-brown-hare-syndrome-virus. Paris: World Organisation Animal Health). Available at: https://www.woah.org/app/uploads/2021/05/european-brown-hare-syndrome-virus-infection-with.pdf (Accessed July 18, 2023)

[ref49] World Organisation for Animal Health (WOAH). (2022). “Rabbit haemorrhagic disease chapter 3.7.2” in WOAH manual of diagnostic tests and vaccines for terrestrial animals 2022. Available at: https://www.woah.org/en/what-we-do/standards/codes-and-manuals/terrestrial-manual-online-access/. (Accessed July 18, 2023)

[ref50] Zanni M. L. Benassi M. C. Scicluna M. T. Lavazza A. Capucci L. (1993). Clinical evolution and diagnosis of an outbreak of European brown hare syndrome in hares reared in captivity. Rev. Sci. Tech. 12, 931–940. doi: 10.20506/rst.12.3.727, PMID: 8219343

