# Supplementary figures and images for: Pathological and virological insights from an outbreak of European brown hare syndrome in the Italian hare (Lepus corsicanus)
Source: Front Microbiol. 2023 Oct 19;14:1250787. doi: 10.3389/fmicb.2023.1250787 (PMC10622795; doi:10.3389/fmicb.2023.1250787)

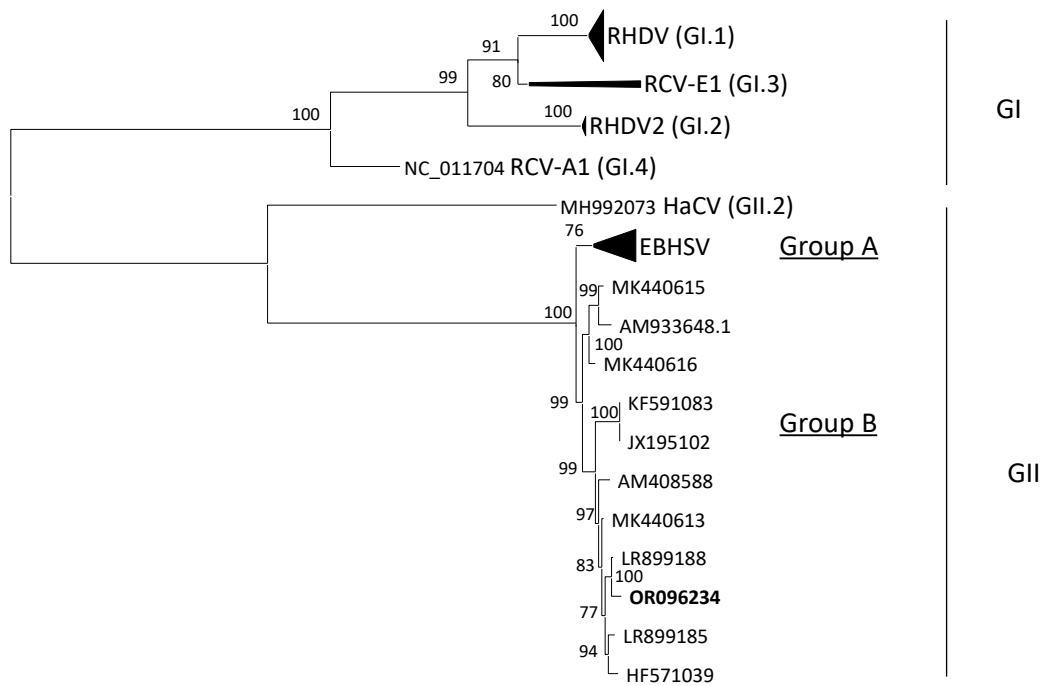

Supplement: Supplementary file 1 [file Data_Sheet_1.PDF]
